# More grateful, less addicted! Understanding how gratitude affects online gaming addiction among Chinese college students: a three-wave multiple mediation model

**DOI:** 10.1186/s40359-023-01271-7

**Published:** 2023-08-23

**Authors:** Bin Gao, Yi Xu, Lu Bai, Gui Luo, Weiyi Li

**Affiliations:** 1 School of Education, Shanghai Normal University, Shanghai, 200234 China; 2 Mental Health Center, Hefei University of Technology, Hefei, 230009 China; 3 School of Business Administration, Jimei University, Xiamen, 361021 China

**Keywords:** Gratitude, Self-control, Loneliness, Online game addiction, College students

## Abstract

**Background:**

Online game addiction has become a prominent public concern, particularly among emerging adults, warranting in-depth investigation. Despite prior cross-sectional research indicating a negative correlation between gratitude and online gaming addiction, a dearth of longitudinal research exists in this area. Furthermore, the underlying mechanisms that explain the link between gratitude and online gaming addiction remain poorly understood, highlighting a critical research gap in the field.

**Methods:**

To bridge this gap, our study adopted a three-wave longitudinal design and constructed a multiple mediation model. Over the course of one year, data was collected from a sample of Chinese undergraduates, with 319 students participating at Time 1, 305 at Time 2, and 292 at Time 3. Participants were administered online self-report surveys, enabling the acquisition of valuable data regarding their levels of gratitude, online game addiction, self-control, and loneliness.

**Results:**

The findings revealed a negative correlation between gratitude measured at Time 1 and online game addiction assessed at Time 3. Further analysis demonstrated that both self-control and loneliness played multiple mediating roles at Time 2 in the link between gratitude and online game addiction.

**Conclusion:**

These research findings shed light on the underlying mechanisms between gratitude and online game addiction, which provide implications for developing interventions (e.g., interventions based on gratitude) for reducing young adults’ online game addiction.

## Introduction

Playing online games is a popular leisure activity, and the number of players worldwide is increasing dramatically. For instance, as of June 2022, the number of online game users in China has reached 552 million, representing approximately 52.6% of all Internet users [1]. However, excessive online gaming may lead to online game addiction. Online game addiction is defined as the persistent and repeated use of the internet to play games that causes significant impairment or distress in a given user’s life [2]. Currently, it is important to acknowledge that online gaming addiction is a broad term that is commonly used to describe excessive and problematic gaming behavior specifically related to online games and lacks standardized diagnostic criteria, unlike internet gaming disorder, which is generally recognized as a clinical condition [3]. Thus, this study adopts the term “online gaming addiction” instead of “internet gaming disorder”. Based on a systematic review, the prevalence rates of online game addiction in China were found to range from 3.5 to 17%, indicating a higher prevalence compared to global reports [4]. Furthermore, online game addiction becomes more prevalent among children and young adults due to the COVID-19 pandemic [5–7]. More importantly, online game addiction could be linked to various detrimental consequences, such as problems in emotional expression [8], poor sleep quality [9], depression [10], and even suicide [11]. Therefore, it is theoretically and practically necessary to investigate the protective or risk factors of online game addiction. In recent years, with the flourishing of positive psychology, gratitude has been increasingly highlighted by researchers for its positive effects on coping with online game addiction [12].

Previous empirical studies have made progress in exploring the correlation between gratitude and online game addiction [13]. However, these studies have two major limitations. Firstly, the prior researcher primarily used cross-sectional designs, limiting the possibility of making causal inferences regarding the relationship between gratitude and online game addiction [12]. Additionally, little empirical evidence exists regarding the underlying mechanisms of this relationship [13]. Secondly, the majority of research in this field has focused on children [14, 15] and adolescents [16–18], neglecting the fact that college students are also at risk for online game addiction [19]. To address these gaps, the present study aims to investigate the following research questions:

1. What is the longitudinal association between gratitude and online gaming addiction among college students?
2. What potential mediating variables exist between college students’ gratitude and their online game addiction?

By examining these research questions, we seek to gain a deeper understanding of the longitudinal relationship between gratitude and online gaming addiction among college students, as well as the potential mediating roles of self-control and loneliness. According to the Interaction of Person-Affect-Cognition-Execution (I-PACE) model [20], the development of addictive behaviors (e.g., online game addiction) is influenced by various factors, including personal characteristics (e.g., trait gratitude), affective states (e.g., loneliness), and behavioral execution (e.g., impaired self-control). Empirical studies have shown that loneliness has been identified as a risk factor for online game addiction [18], while gratitude and self-control are recognized as protective factors against online game addiction [12, 14]. In response to the need for further expansion of the I-PACE model, we aim to integrate these three factors to provide a more comprehensive framework for understanding their combined influences on online game addiction.

### Gratitude and online game addiction

Gratitude refers to an individual’s psychological tendency to recognize and appreciate positive experiences or outcomes resulting from others’ help or kindness, expressed through cognitive, emotional, and behavioral responses [21]. According to the broaden-and-build theory [22], positive emotions like gratitude can foster the development of long-lasting personal resources, such as resilience, that may reduce the likelihood of developing addictive behaviors. Empirical study has shown that gratitude can serve as a protective factor against a range of undesirable addictive behaviors, including substance addiction [23, 24], internet gaming disorder [25], and online game addiction [3]. Furthermore, gratitude has been recognized as a valuable tool for enhancing well-being in 12-step addiction recovery programs, and research suggests that individuals with higher levels of gratitude have positive associations with 12-step practices, post-traumatic growth, and social support, and negative associations with stress and health symptoms [26]. Gratitude interventions have also proven beneficial for individuals with substance use disorders [27, 28]. Therefore, we hypothesize that gratitude would be negatively associated with online game addiction (H1).

### Self-control as a mediator

Self-control pertains to the capacity to regulate one’s thoughts, emotions, and actions in order to attain long-term objectives and effectively respond to evolving situational requirements [29]. Trait gratitude has been found to be positively associated with self-control among university students [30]. According to the strength model of self-control, self-control is viewed as a finite resource that can be gradually exhausted over time [31], while gratitude-based interventions, such as gratitude journaling, can enhance self-control resources [32]. In addition, college students with higher levels of gratitude are more likely to use proactive self-control strategies to avoid temptation [33], which in turn may help curb addictive behaviors. A cross-sectional study has showed that gratitude positively predicts self-control in college students [34]. In addition, self-control serves as a safeguard against online game addiction. For example, a study on interventions for online game addiction found a significant association between increased self-control among gamers and reductions in both the severity of addiction and the amount of time spent playing online games [35]. People with higher levels of self-control showed lower levels of pathological gaming [36] and internet gaming disorder [14]. Moreover, previous research has identified self-control as a mediator in the processes of online game addiction [37]. Thus, we propose that self-control may mediate the link between gratitude and online game addiction (H2).

### Loneliness as a mediator

Loneliness is a negative emotional state that arises when an individual’s social connections fail to meet their expectations, resulting in negative psychological experiences such as emptiness, boredom, helplessness, and bitterness [38]. Young adults have been particularly vulnerable to loneliness and related mental health problems during the COVID-19 pandemic [39, 40]. As a negative emotion, loneliness plays a mediating role in the link between gratitude and mental health problems [41]. In addition, a longitudinal study showed that loneliness could mediate the relationship between parental loneliness and adolescents’ online game addiction [18]. Intervention studies have shown that gratitude-based interventions, such as gratitude writing exercises, can alleviate loneliness in older adults [42]. Moreover, dispositional gratitude is negatively associated with loneliness [43]. Studies have found a positive association between loneliness and online game addiction among adolescents [44], and that gratitude can predict feelings of loneliness among college students [45], while loneliness predicts online game addiction in adolescents [18]. Therefore, it is hypothesized that loneliness may mediate the link between gratitude and online game addiction (H3).

### The serial mediating roles of self-control and loneliness

The I-PACE model posits that individuals with behavioral execution deficiency [20], such as low self-control, and negative affective states like loneliness are more susceptible to developing addictive behaviors, such as online game. Low self-control has also been linked to loneliness, with studies showing that individuals with low self-control may struggle to establish and maintain social relationships, contributing to feelings of loneliness [46]. Similarly, a recent study found that self-control significantly predicts feelings of loneliness among college students [47]. Building on these theoretical and empirical findings, we suggest that gratitude could enhance self-control in young adults, which could help alleviate feelings of loneliness and reduce addictive online game playing in the long term. Consequently, we propose the hypothesis that there may exist a sequential mediation effect of self-control and loneliness in the link between gratitude and online game addiction (H4).

### The present study

**Fig. 1:**
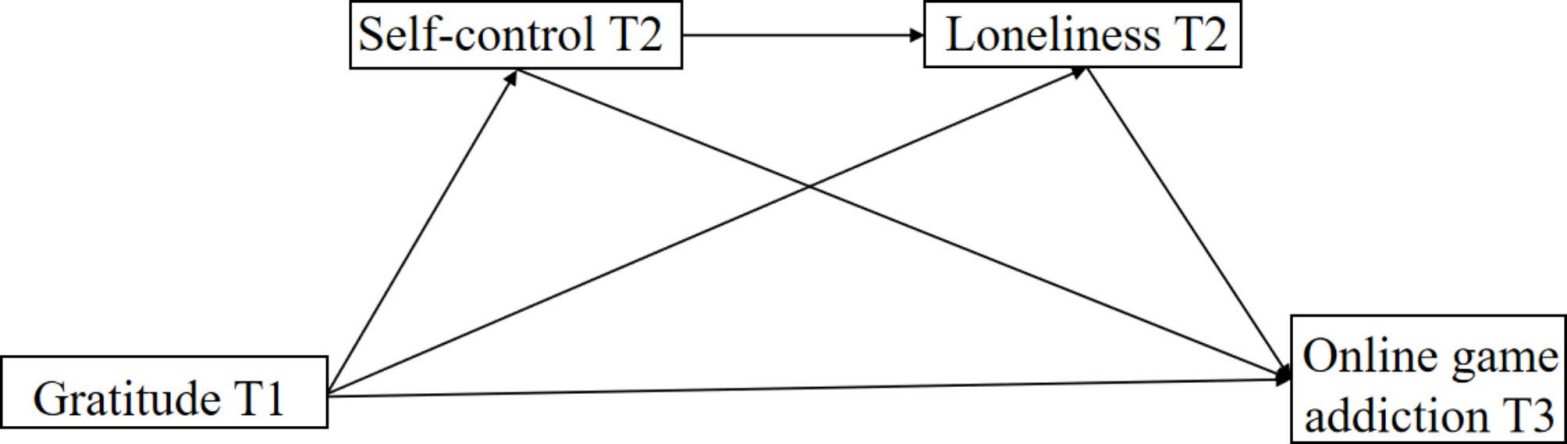
The hypothesized model

In summary, this research offers several significant contributions to the field of online game addiction and also extends the existing literature. Firstly, it investigates the longitudinal association between gratitude and online game addiction, extending previous studies using a cross-sectional study design. Secondly, it employs a three-wave longitudinal design to explore the mediating mechanisms through which gratitude influences students’ online game addiction. Thirdly, it expands the scope of the I-PACE model by incorporating protective factors, such as gratitude, to better explain addictive behaviors. Specifically, the study examines whether the effects of gratitude on online game addiction are mediated by self-control and loneliness (as illustrated in Fig. 1). Notably, this is the first study to directly test this theoretical proposition among college students.

## Method

### Participants

Participants were recruited from a public Chinese university using convenience sampling during the epidemic of COVID-19. This university is a teaching-focused institution with a primary focus on medical disciplines, accommodating a student population exceeding 15,000. The data was collected at three different points in time after the freshmen entered university during the 2021–2022 school year: the beginning (Time1), middle (Time 2), and end (Time 3) of the year. The average interval was 3 months. A total of 381 college freshmen participated in the T1 online survey. Subjects who took less than 5 min to fill out the online questionnaire and had no experience in online games were excluded, resulting in the valid sample of 319 students (M_age_ = 18.24, *SD* = 0.74, 58.9% females). Among the participants, 177 were from urban areas, while 142 were from rural regions. Notably, a majority of the participants were pursuing academic disciplines primarily concentrated in medical-related fields. Of the 319 participants, 305 students and 292 students completed the T2 survey (subject attrition rate = 0.04%) and T3 survey (attrition rate = 0.08%), respectively. All subjects had online game experience at T3, and their self-reported time spent playing online games was 2.56 (*SD* = 1.90) hours per day.

### Procedure

Data for this investigation was collected via the Questionnaire Star Platform, a popular online survey agency in China. Subjects signed an informed consent form before completing the online questionnaire. All participants who were willing to participate in the study signed consent form. They were invited to fill out the surveys in the classroom. This longitudinal study was approved by the ethics committee of the corresponding author’s institution.

### Measures

#### Trait gratitude

The measurement of trait gratitude in this study utilized the gratitude questionnaire–6 developed by McCullough et al. [21]. This unidimensional scale comprises 6 items (e.g., “I am grateful to a wide variety of people”) and has demonstrated good reliability and validity among Chinese college students [30]. Subjects provided their own responses using a 7-point Likert scale, ranging from 1 (*strongly disagree*) to 7 (*strongly agree*). The scores for all items were summed, and the average score was calculated. A higher average score indicates a higher level of gratitude. The Cronbach’s α coefficient for the scale was calculated to be 0.71 at Time 1 in this study.

#### Self-control

In this study, self-control was assessed using the brief self-control scale developed by Morean et al. [48]. This scale comprises 7 items (e.g., “I am good at resisting temptation”) and encompasses two dimensions: self-discipline and impulse control. Previous research has indicated the suitability of this scale for Chinese emerging adults [49]. Notably, items 2, 4, 6, and 7 required reverse scoring. Subjects provided their own responses using a 5-point Likert scale, ranging from 1 (*strongly disagree*) to 5 (*strongly agree*). The average score was obtained by summing the scores for all items. A higher average score indicates a higher level of self-control. The Cronbach’s α coefficient for the scale was calculated to be 0.70 at Time 2 in this study.

#### Loneliness

To assess loneliness in this study, the Short-Form Loneliness Scale developed by Hays and Dimatteo [50] was utilized. This scale consists of eight items that tap into the experience of loneliness, such as “people are around me but not with me”. Previous research conducted among Chinese college students [51] has demonstrated the satisfactory reliability and validity of this unidimensional scale. Participants were instructed to read each item and indicate their responses on a 4-point Likert scale, ranging from 1 (*never*) to 4 (*always*). The scores for all items were summed, and the average score was computed. A higher average score indicated a greater level of loneliness. The Cronbach’s α coefficient for the scale was 0.81 at Time 2 in this study.

#### Online game addiction

The measurement of online game addiction in this study utilized the Chinese version of the Online Game Addiction Scale, which is a subscale derived from the Internet Addiction Scale developed by Zhou and Yang [52]. This scale is a unidimensional measure comprising 8 items (e.g., “I basically spend my time after school playing online games”). Participants responded to these items using a 5-point Likert scale ranging from 1 (*strongly disagree*) to 5 (*strongly agree*). The average score was obtained by summing the scores for all items, with a higher average score indicating a greater level of online game addiction. The Cronbach’s α coefficient for this scale was calculated to be 0.91 at Time 3 in this study.

### Statistical analyses

Data screening was conducted to guarantee the quality of the data. Specifically, we noticed that no outliers were present in the data set, and any missing data were addressed through mean imputation, as the proportion of missing data was less than 1% for all variables [53]. The skewness and kurtosis values (see Table 1) for all variables were assessed, and all values fell within the acceptable range of -2 to + 2 for skewness and − 7 to + 7 for kurtosis, indicating a normal distribution of the data [54]. To test the possible presence of common method biases, we conducted a Harman’s single factor test, which revealed no significant common method variance present in our data. Correlation analyses were performed among the main variables using SPSS 24.0, and we utilized model 6 in the PROCESS macro for the regression analyses. To ensure comparability among variables, all continuous variables were standardized prior to the regression analysis. In addition, gender and age were considered as potential control variables, as prior studies [2, 37] have shown their potential influence on individuals’ online game addiction.

## Results

### Descriptive and correlation analysis

Table 1 displays the descriptive statistics (i.e., mean and standard deviation) and correlation matrix of the variables. Gratitude at Time 1 exhibited a positive correlation with self-control at Time 2, and a negative correlation with loneliness at Time 2 and online game addiction at Time 3. Self-control at Time 2 demonstrated a negative correlation with loneliness at Time 2 and online game addiction at Time 3. Additionally, loneliness at Time 2 exhibited a positive correlation with online game addiction at Time 3. Thus, Hypothesis 1 was supported.

**Table 1:** Descriptive statistics and correlation matrix for key variables

	1	2	3	4	5	6
1.Gender	1					
2.Age	– 0.06	1				
3.Gratitude (T1)	0.18^**^	0.01	1			
4.Self-control (T2)	0.01	– 0.05^*^	0.18^**^	1		
5.Loneliness (T2)	0.05	0.06	– 0.28^**^	– 0.44^**^	1	
6.OGA (T3)	– 0.19^**^	– 0.10	– 0.16^**^	– 0.27^**^	0.26^**^	1
*M* (*SD*)	—	18.25 (0.75)	5.20 (0.89)	3.10 (0.56)	2.01 (0.54)	2.25 (0.80)
Skewness	—	—	– 0.24	0.58	– 0.24	0.16
Kurtosis	—	—	– 0.34	1.73	– 0.09	– 0.73

Note: gender is a dummy variable (0 = male, 1 = female), online game addiction = OGA. ^*^p < 0.05, ^**^p < 0.01

### Testing the multiple mediation model

**Fig. 2:**
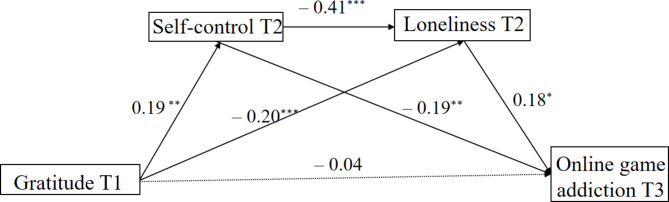
The multiple mediation effects of self-control and loneliness. *Note* The presence of a dashed line in the figures indicates that the path coefficient was not found to be significant.

We employed Model 6 of the PROCESS macro [55] to investigate the multiple mediating effects of gratitude through self-control and loneliness. The results, as presented in Tables 2**and** Fig. 2, demonstrated that all pathways were statistically significant, except for the direct effect of gratitude on online game addiction (β = − 0.04, *p* > 0.05). Specifically, gratitude exhibited a significant positive prediction of self-control (β = 0.19, *p* < 0.01) and a significant negative prediction of loneliness (β = − 0.20, *p* < 0.01). Furthermore, self-control significantly predicted both loneliness (β = − 0.41, *p* < 0.001) and online game addiction (β = − 0.19, *p* < 0.01). Additionally, loneliness had a significant positive effect on online game addiction (β = 0.18, *p* < 0.05). Furthermore, none of the 95% confidence intervals for the path coefficients mentioned above included zero, indicating that all path coefficients were statistically significant.

**Table 2:** Testing the multiple mediation model

Predictors	Model 1 (self-control T2)			Model 2 (loneliness T2)			Model 3 (OGA T3)		
	β	*t*	95% CI	β	*t*	95% CI	β	*t*	95% CI
Gender	–0.14	–1.20	[–0.38, 0.10]	0.15	1.43	[–0.05, 0.37]	–0.37^**^	–3.14	[–0.60, − 0.14]
Age	–0.06	–0.73	[–0.21, 0.10]	0.14^*^	2.05	[0.01, 0.27]	–0.16^*^	–2.25	[–0.31, − 0.02]
Gratitude (T1)	0.19^**^	3.22	[0.07, 0.31]	–0.20^***^	–3.69	[–0.31, − 0.10]	–0.04	–0.82	[–0.17, 0.07]
Self-control (T2)				–0.41^**^	–7.63	[–0.51, − 0.31]	–0.19^**^	–0.30	[–0.32, − 0.06]
Loneliness (T2)							0.18^*^	2.74	[0.05, 0.31]
*R* ^*2*^	0.04			0.26			0.14		
*F*	3.74^**^			23.21^***^			9.11^***^		

Note: online game addiction = OGA, 95% CI = 95% confidence interval, Bootstrap sample size = 5000.

Next, the percentile bootstrap method with bias correction was employed to assess the mediation effect. The results presented in Table 3 indicate that the 95% confidence interval for the direct effect encompasses 0, suggesting non-significance of the direct effect. Conversely, the 95% confidence interval for the indirect path excludes 0, indicating significant mediation effects. These findings suggest that self-control and loneliness serve as complete mediators between gratitude and online game addiction. As a result, Hypotheses 2–4 were supported, indicating significant mediation effects of self-control and loneliness. The multiple mediation model employed in this study accounted for 12% of the variance in online gaming addiction among college students.

**Table 3:** Testing the significance of multiple mediation effects

Model pathways	Effect	*SE*	Bootstrap 95%CI	
			Lower 2.5%	Upper 2.5%
Gratitude → OGA (total effect)	–0.129	0.06	–0.24	–0.01
Gratitude → OGA (direct effect)	–0.042	0.06	–0.15	0.07
Gratitude → Self-control → OGA	–0.037	0.02	–0.08	–0.01
Gratitude → Loneliness→ OGA	–0.036	0.02	–0.08	–0.01
Gratitude → Self-control→ Loneliness → OGA	–0.014	0.01	–0.03	–0.01

Note. OGA = Online game addiction, ^***^p < 0.001, Bootstrap sample size = 5000

## Discussion

This research adopts the I-PACE model as a theoretical framework to examine the influence of predisposing variables (gratitude, self-control, loneliness) on online game addiction among emerging adults, and to provide practical recommendations for addressing the escalating issue of online game addiction. The primary aim of this paper is to contribute to the existing knowledge regarding the longitudinal association between gratitude and online game addiction, as well as to shed light on the underlying mediating mechanisms that link these variables. The present study confirms the presence of a negative relationship between gratitude and online game addiction, and additionally reveals that self-control and loneliness fully mediate this relationship. Further elaboration on the key findings will be provided in the subsequent sections, offering a comprehensive discussion.

The results has shown that gratitude is negatively associated with online game addiction, which is consistent with prior cross-sectional study [12]. Our results are in line with the coping theory [56], which indicates that individuals with high gratitude are more likely to seek instrumental and affective social support from others [57] and they tend to adopt more active coping strategies to deal with problems in life [58], rather than resorting to negative strategies such as online game addiction, thus helping to reduce or counteract online game addiction. Furthermore, this result further supports the broaden-and-build theory proposed by Fredrickson [22]. In detail, gratitude can help people alleviate negative emotions [59] and build personal lasting positive resources, such as self-esteem [60], enhance well-being [61] and life satisfaction [62]. These resources are critical to overcoming game addiction for young people. Accordingly, when such positive resources are diminished or unavailable, individuals are more susceptible to engaging in undesirable behaviors, such as online game addiction.

The findings of this study indicate a negative association between gratitude and online game addiction, which is in accordance with previous cross-sectional research [12]. These results align with the coping theory [56], which suggests that those with greater gratitude are more inclined to seek instrumental and affective social support from others [57]. Moreover, they are more likely to employ active coping strategies to effectively deal with life challenges [58], rather than resorting to negative coping mechanisms like online game addiction. Consequently, gratitude plays a role in reducing or countering online game addiction. Furthermore, these results provide additional support for Fredrickson’s broaden-and-build theory of positive emotions [22]. Specifically, gratitude can assist individuals in alleviating negative emotions [59] and cultivating enduring positive resources such as self-esteem [60], overall well-being [61], and life satisfaction [62]. These personal resources are crucial for combating game addiction among young individuals. Consequently, in the absence of such positive resources, individuals are more susceptible to engaging in undesirable behaviors like online game addiction.

Consistent with our hypotheses, the results indicate that self-control functions as a mediator between gratitude and online game addiction. In the first stage of the mediation process (gratitude → self-control), gratitude significantly and positively predicts self-control, which is in accordance with previous research [34]. There are two plausible explanations for this finding. Firstly, prior studies have demonstrated that people who are grateful are more able to exercise self-control by avoiding temptations [33], delaying immediate gratification, and opting for larger, delayed rewards [63]. This suggests that gratitude may play a vital role in promoting self-control. Secondly, positive emotions like gratitude broaden an individual’s range of thoughts and actions and foster the development of enduring personal resources [64], including resilience and social support. Consequently, individuals are better equipped to manage their emotions and behaviors. In the second stage of the mediation process (self-control → online game addiction), self-control negatively predicts online game addiction, which aligns with previous research [37]. One potential explanation for the link between self-control and online game addiction is that those with greater self-control are more capable of delaying immediate gratification and resisting the immediate rewards associated with addictive behaviors. Instead, they engage in behaviors that offer long-term benefits [65]. In line with self-control theory [29], individuals who possess effective impulse regulation and resistance to temptation are less likely to engage in addictive behaviors, including online game addiction. Hence, it can be inferred that online game addiction may arise from a deficiency in self-control or a failure to exercise self-control.

Consistent with our hypothesis, loneliness is another important explanatory mechanism through which gratitude is linked to college students’ online game addiction. For the first stage of the mediation process (gratitude→loneliness), gratitude could significantly and positively predict loneliness, which is in line with previous studies [34]. Based on a positive psychology perspective, gratitude is suggested to have the potential to alleviate the negative impact of loneliness by fostering psychological flexibility, engagement in life, and strengthening social relationships [66, 67]. By focusing on the positive actions of others and expressing gratitude, individuals may be more likely to form and maintain positive social relationships, which can help to reduce loneliness [68]. For the second stage of this mediation process (loneliness →online game addiction), loneliness could positively predict online game addiction, which is in line with previous studies [37]. One theoretical perspective that may help to explain this finding is the self-medication hypothesis [69, 70], suggesting that individuals may self-medicate or alleviate negative emotions, including loneliness, by engaging in addictive behaviors such as online gaming.

The aim of this study was to investigate the mechanisms that underlie the link between gratitude and online game addiction in college students, with a specific focus on the serial mediating roles of self-control and loneliness. The findings revealed that both self-control and loneliness played individual and combined mediating roles in the link between gratitude and online game addiction. Specifically, gratitude was positively linked to self-control and negatively linked to loneliness, which subsequently influenced the likelihood of experiencing online game addiction among college students. Moreover, the sequential mediation analysis demonstrated that self-control and loneliness sequentially mediated the influence of gratitude on online game addiction. This suggests that higher levels of gratitude promote self-control, leading to reduced loneliness and ultimately decreasing the risk of online game addiction. Overall, This study helps to understand the relationship between the association between gratitude and online game addiction by proposing an integrated sequential mediation model that incorporates gratitude, self-control, loneliness, and online game addiction. This model offers a comprehensive framework that illuminates the underlying mechanisms involved in the link between gratitude and online game addiction.

### Implications

Based on the I-PACE model, we constructed a multiple mediation model to reveal how gratitude affects online game addiction, which has implications for intervening with students addicted to online gaming. First, the current study has expanded existing studies by revealing the longitudinal relationship and the mediation mechanisms between gratitude and online game addiction. Second, to our knowledge, this is the first attempt to reveal that self-control and loneliness are both vital factors linking gratitude to online game addiction, and this result suggests that gratitude intervention programs (i.e., gratitude journaling or letter) can be used to inhibit college students’ online game addiction. Third, previous studies mainly focused on risk factors of online game addiction (e.g., sensation seeking, anxiety and impulsivity), while studying online game addiction from a positive psychology perspective may help identify key character strengths (e.g., gratitude) that could protect college students from developing online game addiction. The I-PACE model currently does not include protective factors (e.g., gratitude) of addictive behavior. Our research results indicate that online compulsive buying are influenced by both risk and protective factors, thus extending the I-PACE model to some extent.

### Limitations and future directions

While our research has provided valuable insights, it is important to acknowledge several limitations. Firstly, this longitudinal study focused on examining whether gratitude can predict online game addiction among freshmen, but it did not explore the reverse relationship between the two variables. Future research could employ a cross-lagged design to investigate the reciprocal association between gratitude and online game addiction. Secondly, our study sample consisted of Chinese college students, and therefore, caution should be exercised when generalizing the findings to other population groups. Additionally, the absence of comparative studies examining online game addiction through cross-cultural and cross-national research is a noteworthy gap. It is crucial for future studies to validate our findings in diverse cultural contexts. Furthermore, we did not distinguish the types of online games that participants played. However, future studies have the opportunity to delve deeper into this aspect and explore how specific game characteristics, such as massively multiplayer online games or role-playing games, may influence the relationship between gratitude and online game addiction. Thirdly, this study primarily explored the link between trait gratitude and online game addiction. It is worth noting that gratitude can be differentiated into two types: trait gratitude and state gratitude. These distinct forms of gratitude may exert varying influences on online game addiction, which could be considered in further research. Lastly, it is essential to assess the effectiveness of gratitude-based interventions in mitigating online game addiction. Future research could employ a quasi-experimental design to examine the effectiveness of gratitude intervention on online game addiction.

## Conclusion

Online game addiction has become a prominent public concern, particularly among college students. Previous research has established a robust correlation between gratitude and online game addiction. We conducted a longitudinal study involving Chinese college students and identified the mediating roles of self-control and loneliness in the link between gratitude and online game addiction. Our findings contribute to a deeper understanding of the mechanisms that connect gratitude and online game addiction, emphasizing the protective influence of gratitude against the development of online game addiction. These findings have significant implications for developing effective coping strategies to promote rational online game playing among college students, as well as for the prevention and intervention of online gaming addiction, such as implementing gratitude-based interventions.

## Data Availability

The corresponding author can provide the datasets used and/or analyzed during the current study upon reasonable request.
